# Chemotherapy impairs ovarian function through excessive ROS-induced ferroptosis

**DOI:** 10.1038/s41419-023-05859-0

**Published:** 2023-05-24

**Authors:** Shenghui Zhang, Qin Liu, Mengyuan Chang, Ying Pan, Badrul Hisham Yahaya, Yanli Liu, Juntang Lin

**Affiliations:** 1 Stem Cell and Biotherapy Technology Research Center, Henan Joint International Research Laboratory of Stem Cell Medicine, Xinxiang Medical University, Xinxiang, China; 2 Department of Biomedical Sciences, Advanced Medical and Dental Institute (IPPT), Universiti Sains Malaysia, Penang, Malaysia; 3 The Third Affiliated Hospital of Xinxiang Medical University, Xinxiang, China

**Keywords:** Chemotherapy, Cell death

## Abstract

Chemotherapy was conventionally applied to kill cancer cells, but regrettably, they also induce damage to normal cells with high-proliferative capacity resulting in cardiotoxicity, nephrotoxicity, peripheral nerve toxicity, and ovarian toxicity. Of these, chemotherapy-induced ovarian damages mainly include but are not limited to decreased ovarian reserve, infertility, and ovarian atrophy. Therefore, exploring the underlying mechanism of chemotherapeutic drug-induced ovarian damage will pave the way to develop fertility-protective adjuvants for female patients during conventional cancer treatment. Herein, we firstly confirmed the abnormal gonadal hormone levels in patients who received chemotherapy and further found that conventional chemotherapeutic drugs (cyclophosphamide, CTX; paclitaxel, Tax; doxorubicin, Dox and cisplatin, Cis) treatment significantly decreased both the ovarian volume of mice and the number of primordial and antral follicles and accompanied with the ovarian fibrosis and reduced ovarian reserve in animal models. Subsequently, Tax, Dox, and Cis treatment can induce the apoptosis of ovarian granulosa cells (GCs), likely resulting from excessive reactive oxygen species (ROS) production-induced oxidative damage and impaired cellular anti-oxidative capacity. Thirdly, the following experiments demonstrated that Cis treatment could induce mitochondrial dysfunction through overproducing superoxide in GCs and trigger lipid peroxidation leading to ferroptosis, first reported in chemotherapy-induced ovarian damage. In addition, N-acetylcysteine (NAC) treatment could alleviate the Cis-induced toxicity in GCs by downregulating cellular ROS levels and enhancing the anti-oxidative capacity (promoting the expression of glutathione peroxidase, GPX4; nuclear factor erythroid 2-related factor 2, Nrf2 and heme oxygenase-1, HO-1). Our study confirmed the chemotherapy-induced chaotic hormonal state and ovarian damage in preclinical and clinical examination and indicated that chemotherapeutic drugs initiated ferroptosis in ovarian cells through excessive ROS-induced lipid peroxidation and mitochondrial dysfunction, leading to ovarian cell death. Consequently, developing fertility protectants from the chemotherapy-induced oxidative stress and ferroptosis perspective will ameliorate ovarian damage and further improve the life quality of cancer patients.

## Introduction

Although aggressive treatments with chemotherapy are routinely performed in cancer patients to prevent the recurrence and metastasis of cancer cells effectively, a substantial number of long-term female survivors have to face ovarian damage from the different cancer treatment modalities, which seriously affect their life quality [1, 2]. The published study reported that the incidence of amenorrhea was 61% in patients younger than 40 years old and 95% in patients older than 40 who received the chemotherapeutic drugs cyclophosphamide (CTX), methotrexate, and 5-fluorouracil [3]. Similarly, the density of non-growth phase follicles in Hodgkin’s lymphoma patients increased after adriamycin, bleomycin, vinblastine, and dacarbazine treatment, and only 1.2% of follicles developed to the second stage after in vitro culture [4]. Moreover, a prospective study evaluated the long-term effects of a CHOP regimen (CTX, doxorubicin, Dox; vincristine and prednisone) on ovarian function in aggressive non-Hodgkin lymphoma [5]. The results indicated that although most patients recovered fertility after chemotherapy, the last menstruation in chemotherapeutic patients was significantly earlier than that in the general population. Therefore, more attention should be paid to chemotherapy-induced reproductive dysfunction to improve female cancer patients’ life quality further.

Generally, cytotoxic chemotherapeutic drugs include alkylating agents, platinum, and anthracyclines. Alkylating agents such as CTX are considered the most gonadotoxic drugs by interfering with DNA replication and inhibiting cell division and proliferation, leading to cell death [6]. Platinum compounds such as cisplatin (Cis) belong to cell cycle non-specific drugs and exhibit similar cytotoxicity as alkylating agents, which can bind to intracellular proteins to exert cytotoxicity by destroying the development of both primordial follicles and growing follicles [7]. Anthracyclines such as Dox can target topoisomerase II and affect the transformation of DNA superhelix into a relaxed state, thereby hindering DNA replication and transcription [8]. Microtubule depolymerization inhibitors such as paclitaxel (Tax) can also stabilize and enhance tubulin polymerization, preventing microtubule depolymerization and resulting in cell mitosis inhibition and cell death [9]. Although the two types mentioned above of chemotherapeutic drugs exhibit a mild or moderate degree of gonadal toxicity, they tend to result in severe ovarian damage in a dose and time-dependent manner.

In particular, the ovary is unique in governing menstruation, hormonal balance, bone metabolism, and fertilization [10]. As an indispensable component of the ovary, the granulosa cells (GCs) surrounding the oocyte experience the periodical morphological changes from flat to cubical and from single to multilayered cells during the reproductive cycle, in which the growth hormones and steroid hormones are secreted by GCs to maintain the oocyte microenvironment, promote follicular development, oocyte maturation and ovulation. Therefore, the damage to GCs may lead to oocyte death, follicular atresia, oligomenorrhea, or even amenorrhea, which negatively affect reproductive function [11–14]. As estimated, chemotherapy drugs are likely to cause follicle atresia by directly inhibiting the proliferative capacity of GCs due to the rapid proliferation of GCs in the process of follicle development and further lead to premature activation of primordial follicles and early depletion of the follicle pool [15, 16]. Previous studies have demonstrated that chemotherapy-induced ovarian damage may result in various ways, mainly manifested as but not limited to atresia of growing follicles, excessive activation of primordial follicles, interstitial fibrosis, and acute ovarian vascular toxicity [17]. However, the underlying mechanism of chemotherapeutic drugs-induced ovarian dysfunction remains unclear, and exploring this underlying mechanism will provide a basement for developing protective agents to preserve the normal reproductive function in female patients during conventional cancer treatment.

Consequently, we first analyzed the abnormality of sex hormones in female cancer patients after chemotherapy and further confirmed the gonadal toxicity of four types of conventional chemotherapeutic drugs (CTX, Cis, Dox, and Tax) in vivo. Subsequently, based on the published reports and RNAseq data, we focused on the oxidative stress and ferroptosis that play critical roles in chemotherapeutic drugs-induced ovarian dysfunction, especially revel their potential roles in Cis-induced viability loss of GCs. Based on our harvested results, we strongly recommend considering the chemotherapy-induced ovarian damage during conventional cancer treatment for female patients and providing support for screening and applying fertility-protective adjuvants, contributing to the improvement of their life quality.

## Materials and methods

### Patients

This study was approved by the Ethics Committee of the Xinxiang Medical University, and the participants were premenopausal women aged <45 years with regular spontaneous menstruation diagnosed with breast cancer. These patients were recruited from the First Affiliated Hospital of Xinxiang Medical University to evaluate their ovarian damage after receiving chemotherapy. The study participants were premenopausal women aged <45 years with regular spontaneous menstruation diagnosed with breast cancer. These patients were recruited to evaluate their ovarian damage after receiving chemotherapy. Chemotherapy regimens were Dox-CTX-based protocols followed by Tax or Tax-based treatment plus trastuzumab. The control group consisted of 10 healthy female volunteers aged between 30 and 45. Serum samples were collected from patients between 1 and 3 years after chemotherapy, and then the follicle-stimulating hormone (FSH), estradiol (E2), and luteinizing hormone (LH) levels were clinically determined (Table S1). Written informed consent was obtained from all patients and donors prior to serum collection, and all patients and donors provided consent for the use of their serum samples for scientific research.

### Animals and treatments

Female ICR mice (18–25 g) were purchased from Vital River Laboratory Animal Technology (Beijing, China), and all animal experiments adhered to the ARRIVAL guidelines and were performed following the National Research Council Guide for the Care and Use of Laboratory Animals and have been licensed by the Ethics Committee of Xinxiang Medical University.

In the first stage, the mice were randomly divided into five groups (*n* = 10): Control group, CTX (Endoxan, Germany) treated group (150 mg/kg, i.p), Cis (Yunnan Phytopharmaceutical, China) treated group (15 mg/kg, i.p), Dox (Lunan Pharmacy, China) treated group (12 mg/kg, i.p), Tax (Baisainuo, China) treated group (35 mg/kg, i.p), and the dosage selection of agents referred to clinical drug use guidelines for administration. During the experimental period, the mice in each group respectively received chemotherapeutic drugs once a week for consecutive 4 weeks. At the end of the experiment, the survival rates of mice in each group were recorded, and the serum and ovarian samples were isolated for the subsequent experiments. The serum samples were sent to Xinxiang Assegai Medical Laboratory Center (Xinxiang, China) within 8 h, and the activity of associated enzymes (ALT, AST, and GGT) was determined by velocity method; the content of Urea, UA, and Creatinine (CRE) was quantified by dehydrogenase and oxidase methods.

In the second stage, the mice were randomly divided into two groups (*n* = 10): Cis treated group (15 mg/kg, i.p) and the Cis + N-acetylcysteine (NAC, Aladdin, A105422, China) treated group. In the Cis + NAC treated group, the mice received Cis treatment (15 mg/kg, i.p.) as mentioned above, and then mice were orally given NAC solved in drinking water at a dose of 10 mg/ml for 3 months. At the end of the experiment, the ovarian samples of mice were isolated for subsequent experiments. The investigator was blinded to the group allocation of the animals during the experiment. No statistical method was used to predetermine the sample size for the mice experiment, which was based on preliminary experimental results. The sample size of each experiment is shown in the legend. No data were excluded from the analysis.

### Cell culture

The SVOG and KGN human ovarian granulosa cell lines were purchased from OTWO Biotechnology Co., Ltd (HTX2650, HTX2045, China) and were cultured in high-glucose DMEM (ZQ-121; ZQXZBIO, China) containing 10% (v/v) fetal bovine serum and 1% (v/v) penicillin/streptomycin at 37 °C with 5% CO_2_. When the cell density reached 90% confluence, the cells were subcultured. Both SVOG and KGN cells have been authenticated using STR profiling.

### Hematoxylin and eosin (HE) staining

HE staining was performed following a routine procedure. Briefly, paraffin sections (3 μm) were deparaffinized and rehydrated, then stained with hematoxylin and eosin (Solarbio, G1120, China). After dehydration with graded alcohol and clearing in xylene, the mounted slides were examined, sealed, and imaged under a microscope (Nikon, Japan).

### Masson staining

According to the manufacturer’s instructions (Solarbio, G1346, China), paraffin sections were subjected to Masson staining. The level of ovarian fibrosis was evaluated by calculating the percentage of the blue staining area (collagen).

### TUNEL staining

After full deparaffinization and hydration, the sections were incubated with 20 μg/ml of proteinase K for 20 min, washed with PBS, and incubated with 50 μl of TUNEL assay solution (Servicebio, G1501, China) for 1 h at 37 °C in the dark. Subsequently, the sections were sealed with an anti-fluorescence quenched solution and imaged under a fluorescence microscope (Nikon, Japan).

### Immunohistochemistry

Paraffin sections were dewaxed and soaked in H_2_O_2_ solution for 15 min; after antigen retrieval for 10 min, the samples were treated with blocking solution for 30 min and incubated with primary antibody AMH overnight at 4 °C; then the samples were incubated with a secondary antibody for 1 h at room temperature, and were stained with 3,3′-diaminobenzidine (DAB) followed by hematoxylin for 5 min. The samples were washed in PBS 3 times between each step. Finally, the sections were sealed and imaged under a microscope (Nikon, Japan).

### CCK8 assay

Cells were seeded into a 96-well plate (3 × 10^3^ cells/per well) and cultured overnight. Then, the storing solution of Tax (final concentration: 0, 2.5, 5, 10, 20 μg/ml) or Cis (final concentration: 0, 10, 25, 50, 100 μg/ml) was added to each well, and the cells were cultured for another 24 or 48 h. Then, 5 μl of CCK-8 solution (Beyotime, C0038, China) was added into each well, and after incubating for 3 h, the absorbance at 450 nm was determined using a microplate reader (Thermo, USA).

### Apoptosis assay

Cells were treated with 5 μg/ml of Tax or 25 μg/ml of Cis for 24 h, and then the cells were digested and gently re-suspended in a staining buffer containing 5 μl Annexin V-FITC storing solution and 10 μl storing propidium iodide solution according to the instructions of Annexin V-FITC detection Kit (Beyotime, C1062S, China); after that, the cells were incubated for 20 min in the dark at 4 °C. At the end of incubation, the cells were detected by flow cytometry (BD FACSCalibur, USA).

### Calcein AM/PI assay

Cells were treated with different concentrations of Dox (final concentration: 0, 10, 25, 50, 100 μg/ml) for 24 h. After removing the culture medium, the cells were washed with PBS twice, and 250 μl of Calcein AM/PI assay working solution (Beyotime, C2015S, China) was added to each well incubated at 37 °C in the dark for 30 min. After that, the cells were observed and imaged under an inverted fluorescence microscope (Leica, German).

### Intracellular reactive oxygen species (ROS) assay

The intracellular ROS levels were measured using a ROS assay kit (C1300-1, Applygen, China). Cells were respectively exposed to different concentrations of Tax (final concentration: 0, 2.5, 5, 10, 20 μg/ml), Cis (final concentration: 0, 10, 25, 50, 100 μg/ml), or 25 μg/ml of Cis with and without NAC (5 mM) for 24 h. Then, the cells were stained with 5 μM of DCFH-DA at 37 °C for 30 min, and the fluorescence-labeled cells were further analyzed by flow cytometry.

### Mitochondrial membrane potential (MMP) assay

The JC-1 staining kit (Abcam, ab113850, England) was used to measure the MMP of cells. Briefly, the cells were incubated with Tax (5 μg/ml), Dox (10 μg/ml), or Cis (25 μg/ml) for 24 h. Afterward, the cells were incubated in JC-1 working solution (25 μM) for 30 min at 37 °C in a dark environment, and then the fluorescence-labeled cells were further observed and imaged under an inverted fluorescence microscope.

### Mitochondrial transmembrane potential (ψm) assay

TMRE indicator (Beyotime, C2001S, China) was used to detect mitochondrial transmembrane potential (ψm) according to the manufacturer’s protocol. Briefly, the cells were treated with 25 μg/ml of Cis for 1, 2, 4, 8, and 24 h and were further incubated with TMRE working solution (10 μM) at 37 °C for 30 min, and then, the fluorescence-labeled cells were imaged under inverted fluorescence microscopy and analyzed by flow cytometry.

### Mitochondrial ROS assay

MitoSOX^TM^ Red mitochondrial superoxide indicator (Invitrogen, M36008, USA) was used to detect the mitochondrial ROS by selectively targeting mitochondria in cells. Briefly, the cells were incubated with 25 μg/ml of Cis for 12 h, followed by treatment with 5 μM of MitoSOX working solution for 20 min, and then the fluorescence-labeled cells were subjected to flow cytometry.

### Western blot

Cells were incubated with Tax (5 μg/ml), Dox (10 μg/ml), Cis (25 μg/ml), or Cis (25 μg/ml) + NAC (5 mM) for 24 h. After treatment, the protein samples of harvested cells and tissues were prepared with RIPA Lysis Buffer (Cwbio, China), and protein concentrations were measured using the BCA Protein Assay Kit (Beyotime, China). SDS-PAGE was used to separate proteins before being transferred to polyvinylidene difluoride membranes (Millipore), and the membrane with targeted proteins was then blocked for 1 h at room temperature in 5% nonfat milk, followed by overnight incubation with primary antibody (Table S2) at 4 °C. After washed with PBS for three times, the membranes with targeted proteins were incubated with secondary antibody for 1 h at room temperature. Finally, the signals were displayed using the Enhanced chemoluminescence detection kit (Millipore, IPVH00010, Germany) and detected using Amersham Imager 600 (GE Healthcare Life Sciences, USA) with Image Lab software.

### Immunofluorescence

After deparaffinization and hydration, the tissue sections were heated in a microwave for 45 min for antigen retrieval. Simultaneously, Cells were incubated with Tax (5 μg/ml), Dox (10 μg/ml), and Cis (25 μg/ml) for 24 h and fixed in 4% paraformaldehyde at room temperature for 15 min. Then, tissue and cell samples were permeabilized and blocked with 3% BSA, and 0.03% Triton X100 for 1 h, followed by overnight incubation with primary antibodies (Table S2) at 4 °C. After being washed with PBS, the samples were incubated with secondary antibodies (Table S2) for 1 h at room temperature, and then Hoechst 33342 (Beyotime, C1027, China) was conventionally used to stain the cell nucleus. Immunofluorescence was detected using a fluorescence microscope. Finally, the samples were observed and imaged under inverted fluorescence microscopy.

### Lipid ROS assay

Cells were respectively treated with Cis (25 μg/ml), Cis + Deferoxamine (DFO, 100 μM), and Cis + NAC (5 mM) for 24 h and then were incubated with 5 μM C11-BODIPY 581/591 (Invitrogen, D3861, China) at 37 °C for 30 min in the dark environment. Finally, the cellular lipid ROS was observed and imaged under inverted fluorescence microscopy.

### Reduced glutathione (GSH), oxidized glutathione (GSSG), and malondialdehyde (MDA) assay

After treatment with Cis (25 μg/ml) for 24 h, cell lysates were first obtained with RIPA Lysis Buffer. The GSH and GSSG level was measured with the GSH and GSSG Detection Kit (Solarbio, BC1175, BC1185, China), and the level of MDA was measured with Lipid Peroxidation MDA Assay Kit (Solarbio, BC0020, China). Finally, a microplate reader was used to determine the GSH and GSSG at 412 nm and MAD by the ratio of 532 nm to 600 nm.

### RNA-sequencing (Seq) analyses

RNA-Seq services were provided by Beijing Genomics Institute (BGI, China). Briefly, RNA extraction of ovarian tissues, Oligo (dT) magnetic bead enrichment, and reverse transcription with N6 random primers were performed by BGI to ensure the high quality of the samples, and then the sequencing was performed by BGI using the BGISEQ-500 platform. After that, the differential expression analysis was performed using the DESeq2 (v1.4.5) with Q value ≤ 0.05, and the screened differentially expressed genes were further analyzed with pathway enrichment based on the KEGG database.

### Transmission electron microscopy (TEM)

The cells treated with Cis (25 μg/ml) for 24 h were fixed using TEM fixative (Servicebio, G1102, China), followed by agarose pre-embedding and 1% O_S_O_4_ post-fixation. Then samples were dehydrated in a graded series of ethanol baths and embedded in resin. 60 nm of Ultrathin sections were stained with 2% uranium acetate and lead citrate and further observed and imaged under TEM (HT7800, HITACHI).

### Statistical analysis

Statistical analysis was performed using GraphPad Prism 8.0 analysis software. Descriptive statistics (mean ± SD) are reported for gonadal hormone levels (FSH, LH, E2). All cell experiments were performed triplicate at least. Significant differences were determined by the independent Student’s *t*-test between the two groups, while one-way ANOVA analysis was performed to compare multiple groups. *P* < 0.05 was considered statistically significant.

## Results

### Abnormal gonadal hormone levels were observed in patients who received chemotherapy

**Fig. 1:**
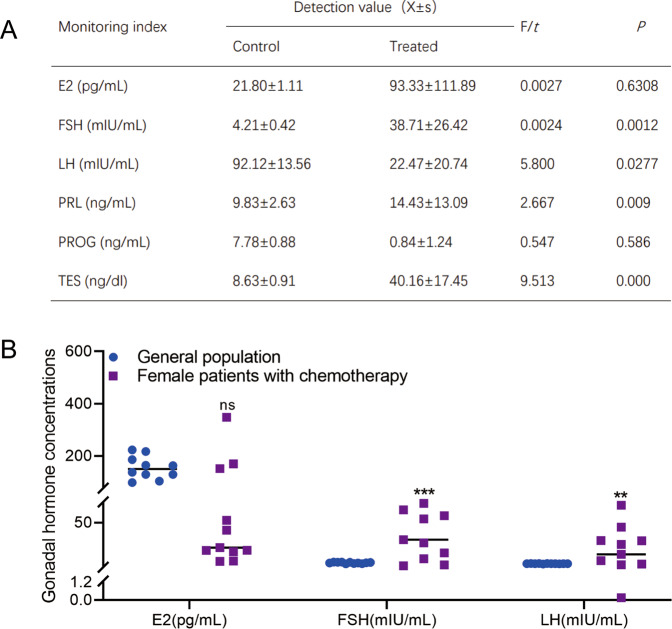
Abnormal gonadal hormone levels in chemotherapy patients. **A**, **B** FSH, E2, and LH levels of chemotherapy patients were determined using chemiluminescence.

The median age of patients who received chemotherapy in this study was 41 (29–44) years. The gonadal hormone levels among the study population are shown in Table S1 and Fig. 1. In comparison with the healthy females, the patients with chemotherapy revealed significant upregulated levels of serum FSH (*P* = 0.0003) and LH (*P* = 0.0099) (Fig. 1). Detailedly, the total frequency of patients with premature ovarian insufficiency (POI) was 70% (7/10). The E2 level of 5 patients (5/10) was less than 30 pg/ml, while the E2 level of the other 3 patients (3/10) was more than 100 pg/ml, and also in these 3 patients, the FSH level was less than 40 mIU/ml (Table S1). Therefore, it is reasonably postulated that the upregulation of the E2 level may result from the disordered growth of follicles in the early stage of POI.

### Chemotherapeutic agents induced acute ovarian injury

**Fig. 2:**
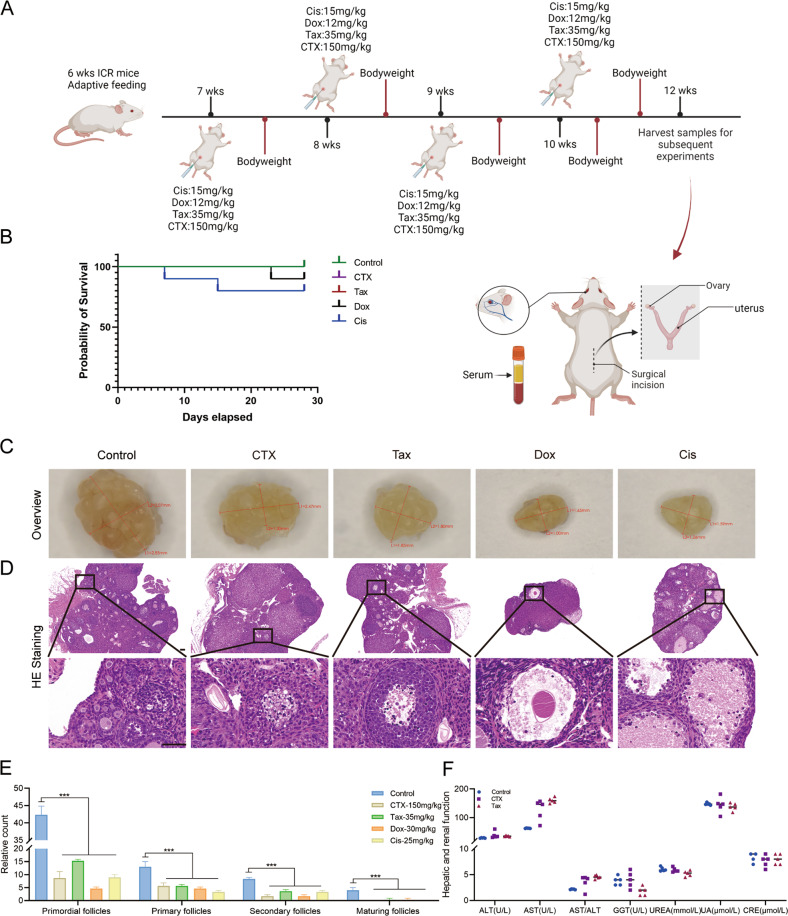
Chemotherapeutic agents induced acute ovarian injury. **A** The flowchart of the experimental procedure. The mice in each group received CTX, Tax, Dox, or Cis, respectively, once a week for four consecutive 4 weeks from 7 to 10 weeks. All mice were sacrificed at 12 week-age. *n* = 10 mice/group. **B** Kaplan–Meier survival curves were used to record the survival of the mice during the agent administration. **C** The ovarian macrostructure was observed under visible light. **D** Representative images of H&E staining of ovarian micromorphology after treatment with chemotherapeutic agents. Scale bars = 100 μm. *n* = 5 (independent experiments). **E** The quantities of primordial, primary, secondary, and maturing follicles per slide in each group were analyzed. *n* = 5 (independent experiments). **F** Conventional biochemical methods were used to detect the activity of levels of ALT, AST, GGT UREA, UA, and CRE in the serum of mice. *n* = 5 (independent experiments). ***p < 0.001.

Four typical chemotherapeutic agents were selected to visually observe the ovarian damage induced by chemotherapy to evaluate their potential gonadal toxicity in animal models. The flowchart of the detailed experimental procedure is displayed in Fig. 2A. As shown in Kaplan–Meier survival curves (Fig. 2B), all mice survived throughout the experiment in the Control, CTX, and Tax treatment groups, while the survival rate was 90% (1/10) in the Dox treatment group and 80% (2/10) in the Cis treatment group.

Subsequently, the macromorphological assessment of the ovary exhibited that the ovarian volume was significantly atrophied after treatment with chemotherapeutic agents, especially in the DOX treatment group (Fig. 2C). Simultaneously, exposure to the chemotherapeutic agents negatively affected the total number of follicles, and the number of primordial follicles which were almost invisible in the ovaries of Cis treated mice. Consistently, the numbers of preantral and antral follicles were significantly decreased, and the maturing follicles were barely observed in the ovaries of mice with chemotherapeutic agent exposure (Fig. 2D, E). Furthermore, the key indicators of liver and kidney function were detected in the serum of CTX and Tax-treated mice, and the significant upregulation of aspartate aminotransferase (AST) level indicated that CTX and Tax treatment resulted in acute liver injury, but no obvious cytotoxicity to kidney (Fig. 2F).

### Chemotherapeutic agents led to ovarian fibrosis and reduced ovarian reserve

**Fig. 3:**
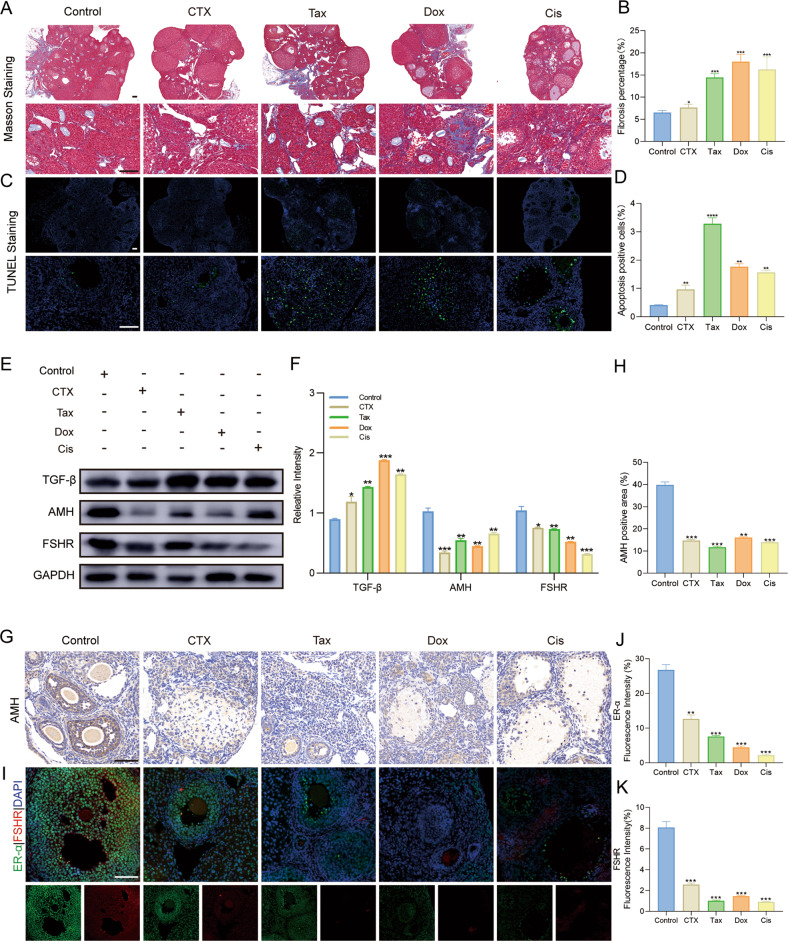
Chemotherapeutic agents induced serious ovarian dysfunction. **A**, **B** Representative images of Masson staining were exhibited to indicate the ovarian stroma fibrosis in mice after treatment with chemotherapeutic agents (**A**) and the further quantification in each group (**B**). Scale bar =100 μm. n = 5 (independent experiments). **C**, **D** Representative images of TUNEL staining in the ovaries of mice were shown (**C**), and the ratio of apoptotic cells was quantified (**D**). Scale bar =100 μm. *n* = 5 (independent experiments). **E**, **F** Expressions of TGF-β, AMH, and FSHR were determined by the Western blot in the ovaries (**E**) and further quantified with Image J software (**F**). GAPDH was used as the internal control. *n* = 3 (independent experiments). **G**, **H** Representative immunohistochemistry images for AMH (**G**) and further semi-quantitative comparisons among the five groups (**H**) were shown. Scale bar = 100 μm. *n* = 3 (independent experiments). **I**–**K** Representative photographs of immunofluorescence (**I**) and semi-quantitative comparisons were shown among the five groups for ER-α (green) (**J**) and FSHR (red) (**K**). Scale bar = 100 μm. *n* = 3 (independent experiments). *p < 0.05, **p < 0.01, ***p < 0.001.

Masson staining was used to determine the effects of chemotherapeutic agents on ovarian stromal fibrosis. As shown in Fig. 3A, B, in the ovaries of CTX, Tax, Dox, and Cis treated mice, the ovarian stroma was disordered and atrophied and accompanied by the significant upregulation of TGF-β (Fig. 3E); while Dox treatment exhibited the maximum toxicity on the ovarian stroma presented as the most obvious fibrosis degree, but CTX treatment exhibited moderate toxicity on the ovarian stroma. Subsequently, ovarian cell apoptosis was examined by TUNEL staining, and green fluorescence-labeled (apoptotic) cells were calculated. As shown in Fig. 2C, D, an abundance of healthy follicles in the ovaries of mice in the control group was observed, and almost no apoptotic cells were recorded. In contrast, masses of the antral follicles in the ovaries of CTX, Tax, Dox, and Cis-treated mice stayed at an atretic state as characterized by the increase of apoptotic cells and the presence of detached granulosa cell layers.

Moreover, the expression of AMH, FSHR, and ER was examined to evaluate the ovarian reserve of mice, as intact reproductive function depends on sufficient ovarian reserve. AMH represents the best marker to assess the decline of ovarian reserve, and the unidirectional rise in FSH and monotropic decline in ER are semaphores of the menopausal transition. As expected, AMH, FSHR, and ER expression was significantly downregulated in CTX, Tax, Dox, and Cis-treated mice, which strongly suggested the poor ovarian reserve and the potential poor response to ovarian stimulation (Fig. 3E–K).

### Chemotherapeutic agents impaired viability of ovarian GCs

Based on the results in vivo, SVOG and KGN cells were selected to detect the potential cytotoxic mechanism of chemotherapeutic agents. Results of CCK-8 assays indicated that cell proliferation was markedly reduced in both time-dependent and dose-dependent manner after Tax and Cis treatment (Fig. S1A, B), and the results of flow cytometry indicated that Tax and Cis treatment could induce the apoptosis of SVOG and KGN cells (Fig. S1C, D). After that, the Dead/Living staining results also confirmed that DOX treatment induced the apoptosis of SVOG and KGN cells in a dose-dependent manner (Fig. S1E–G). Furthermore, Tax, Dox, and Cis treatment significantly downregulated the expression of FSHR in ovarian GCs, which is a crucial facilitator of steroidogenesis and oocyte maturation (Fig. S2A–D).

### Chemotherapeutic agents induced apoptosis of ovarian GCs through oxidative stress

**Fig. 4:**
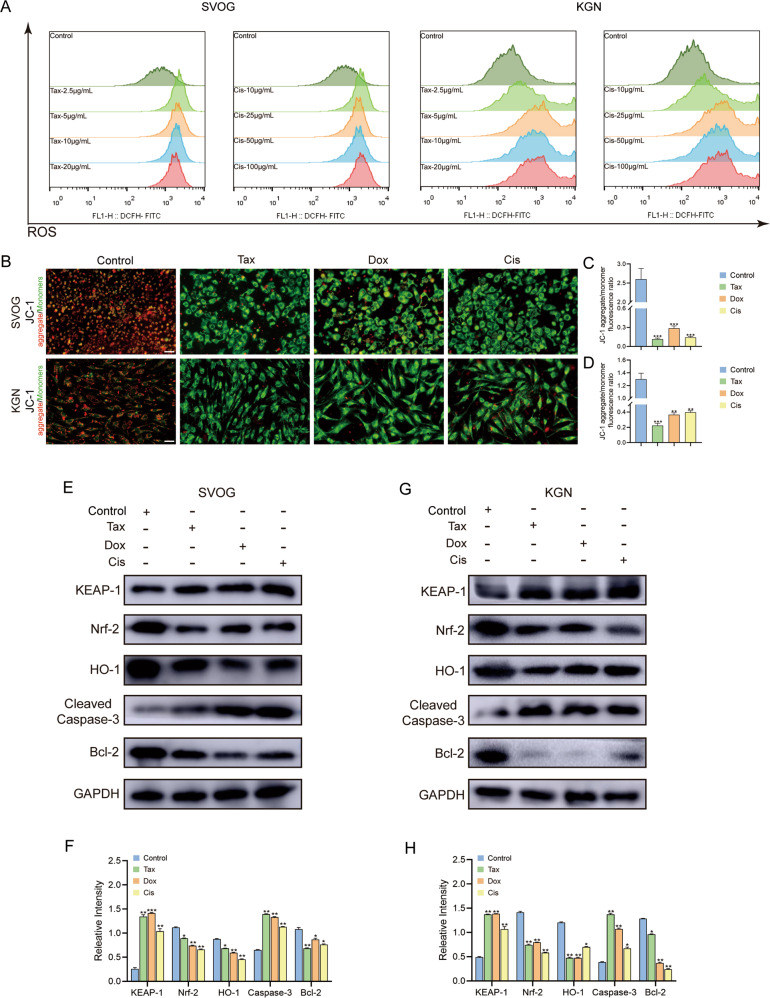
Chemotherapeutic agents induced apoptosis of ovarian GCs through oxidative stress. **A** ROS assay to evaluate the intracellular ROS levels of SVOG and KGN cells after Tax (0, 2.5, 5, 10, 20 μg/ml) or Cis (0, 10, 25, 50, 100 μg/ml) treatments for 24 h. **B**–**D** SVOG and KGN cells were treated with Tax (5 μg/ml), Dox (10 μg/ml), or Cis (25 μg/ml) for 24 h and then stained with JC-1 (red for aggregate, green for monomer). Scale bar = 10 μm. The JC-1 aggregate/monomer fluorescence ratio was quantified for SVOG (**C**) and KGN (**D**) cells. *n* = 3 (independent experiments). **E**, **G** The protein levels of KEAP-1, Nrf-2, HO-1, Cleaved Caspase-3, and Bcl-2 were examined using Western blot analysis in SVOG (**E**) and KGN (**G**) cells. **F**, **H** Quantitative analysis of the targeted protein expressions. *n* = 3 (independent experiments). *p < 0.05, **p < 0.01, ***p < 0.001.

Disturbing the mitochondrial respiratory chain and generating excessive ROS inhibit cancer cell proliferation for various chemotherapy agents [7]. Similarly, Tax and Cis treatment significantly upregulated intracellular ROS levels (Fig. 4A). Generally, the sign of JC-1 aggregates (red) is regarded as the marker of mitochondrial polarization, whereas the cells solely containing JC-1 monomers (green) indicate mitochondrial depolarization. As shown in Fig. 4B–D, Tax, Dox, and Cis treatment impaired the mitochondrial activity by reducing the MMP. Furthermore, we found that the expression of Nrf-2 and HO-1 was significantly downregulated, whereas KEAP-1 was upregulated (Fig. 4E–H). In addition, the expression of Bcl-2 was markedly downregulated in the ovarian GCs following Tax, Dox, and Cis exposure, but cleaved caspase 3 was upregulated (Fig. 4E–H).

### Cis-induced ovarian damage through promoting ferroptosis

**Fig. 5:**
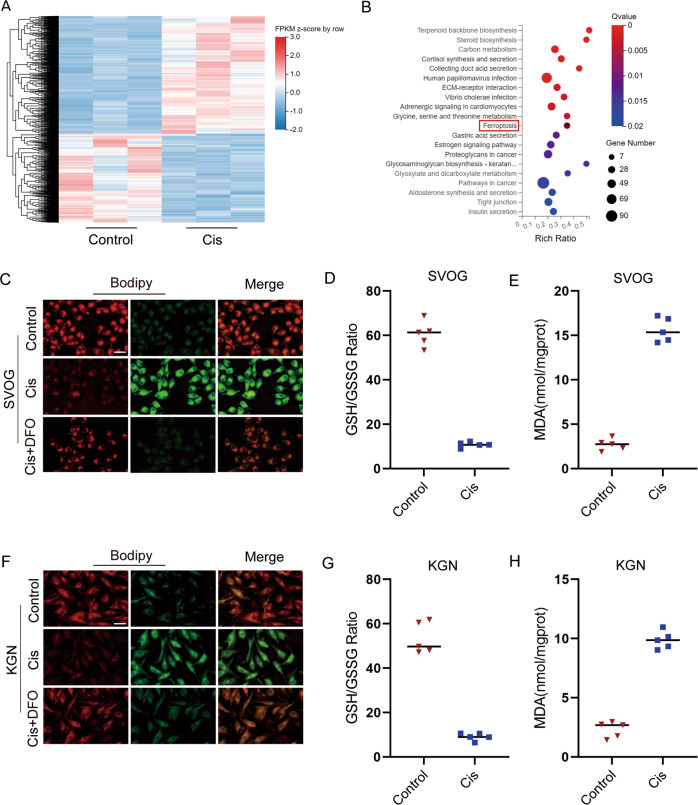
Ferroptosis is involved in Cis-induced ovarian injury. **A**. Heatmap indicated that 2584 genes were identified with significant expression change (2 fold with adjusted *P* < 0.05) after Cis-treated mice. *n* = 3 (independent experiments). **B** Differential pathways enriched in ovaries of Control and Cis-treated mice by KEGG. *n* = 3 (independent experiments). **C**, **F** Representative images of BODIPY staining indicated lipid ROS level after Cis (25 μg/ml) or Cis (25 μg/ml) + DFO (100 μM) treatments (red for reduction, green for oxidization) in SVOG (**C**) and KGN (**F**) cells. Scale bar = 10 μm. *n* = 3 (independent experiments). **D**, **G** The ratio of Intracellular GSH/GSSG was assayed after Cis (25 μg/ml) treatment for 24 h in SVOG (**D**) and KGN (**G**) cells. *n* = 5 (independent experiments). **E**, **H** Intracellular MDA were assayed after Cis (25 μg/ml) treatment for 24 h in SVOG (**E**) and KGN (**H**) cells. *n* = 5 (independent experiments).

RNA-seq was performed between the ovarian tissues of mice with and without Cis treatment to further investigate the underlying mechanism by which Cis induced premature ovarian failure. Strikingly, 2584 genes were identified with significant expression change (2 fold with adjusted *P* < 0.05), and 347 KEGG pathways were significantly identified. Notably, ferroptosis signaling pathways were preliminarily predicted to result in Cis-induced ovarian damage (Fig. 5A, B). Thus, we further validated iconic events related to ferroptosis by monitoring lipid ROS accumulation with C11 BODIPY 581/591. As estimated, after Cis treatment, a significant increase of lipid ROS accumulation was observed in ovarian GCs, but when the cells were treated with the combination of DFO (ferroptosis inhibitors) and Cis, lipid ROS level was markedly reduced (Fig. 5C, F). We also demonstrated that the GSH/GSSG ratio was downregulated in Cis-treated ovarian GCs, but the MDA level increased (Fig. 5D, E, G, H).

### Cis induced ferroptosis through mitochondrial dysfunction and impaired antioxidant capacity

**Fig. 6:**
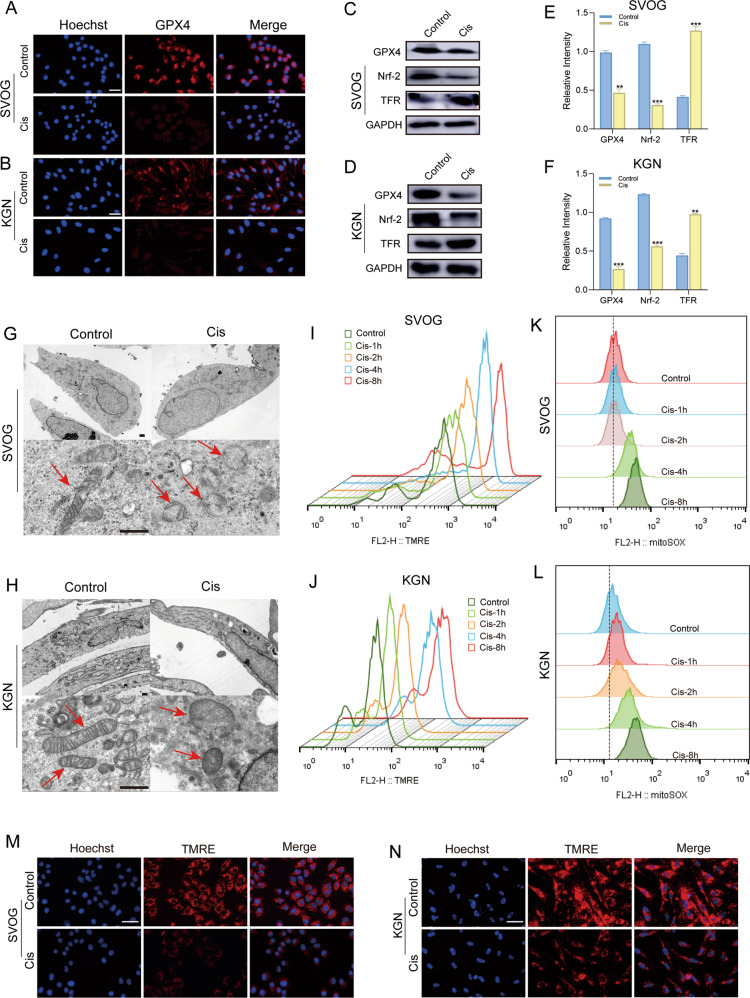
Cis-induced ferroptosis through mitochondrial dysfunction and impaired antioxidant capacity. **A**, **B** Assessment of GPX4 expression in SVOG (**A**) and KGN (**B**) cells treated with Cis (25 μg/ml) for 24 h. Scale bar = 10 μm. **C**, **D** The protein levels of GPX4, Nrf-2, and TFR were examined using Western blot analysis in SVOG (**C**) and KGN (**D**) cells treated with Cis (25 μg/ml) for 24 h. **E**, **F** Quantitative analysis of the targeted protein expressions. *n* = 3 (independent experiments). *p < 0.05, **p < 0.01, ***p < 0.001. **G**, **H** Representative cell and mitochondrial ultrastructural images of SVOG (**G**) and KGN (**H**) cells exposed to Cis (25 μg/ml) for 24 h. Scale bar = 5 μm. **I**, **J** TMRE assay to evaluate the MMP levels of SVOG (**I**) and KGN (**J**) cells after Cis (25 μg/ml) treatment for 1, 2, 4, and 8 h by flow cytometry. **K**, **L** MitoSOX assay to evaluate mitochondrial ROS levels of SVOG (**K**) and KGN (**L**) cells after Cis (25 μg/ml) treatment for 1, 2, 4, 8 h by flow cytometry. **M**, **N** Representative images of TMRE staining indicated MMP levels of SVOG (**M**) and KGN (**N**) cells after Cis (25 μg/ml) treatment for 24 h. **p < 0.01, ***p < 0.001.

GPX4, a key enzyme that reduces intracellular lipid ROS, is imperative for riding ferroptosis, and its deletion is sufficient to induce ferroptosis [18]. As shown in Fig. 6A–F, both immunofluorescence and western blot results demonstrated the significant downregulation of GPX4 in Cis-treated ovarian GCs. Importantly, GPX4 is also a transcription target of Nrf-2. Thus, the interruption of Nrf-2 activation may lead to the downregulation of GPX4. Consistently, the significant downregulation of Nrf-2 in Cis-treated ovarian GCs was also confirmed, while the expression of transferrin receptor (TFR) was upregulated, which strongly suggested that Cis treatment was likely to result in ovarian dysfunction through ferroptosis.

Recently, ferroptosis has been recognized as mitochondria-related cell dysfunction actuated through accelerated ROS levels [19]. Nonetheless, whether the mitochondria play a critical role in the execution of ferroptosis remains highly controversial. Interestingly, our results indicated that with the mitochondria’s polarization, the mitochondrial ROS level was upregulated in Cis-treated ovarian GCs, while the MMP was significantly downregulated. Furthermore, the mitochondrial ultrastructure examined by TEM was shown in Fig. 6G, H, the mitochondria swelled irregularly, and the cristae fractured and became blurred in Cis-treated ovarian GCs when compared with the untreated cells. Consequently, these results indicated that Cis treatment could induce the excessive production of mitochondrial ROS through stimulating MMP hyperpolarization, which provides the necessary conditions for ferroptosis, followed by mitochondrial disintegration and cristae disappearance (Fig. 6I–N).

### NAC inhibited ferroptosis by reducing ROS levels and enhancing antioxidant capacity

**Fig. 7:**
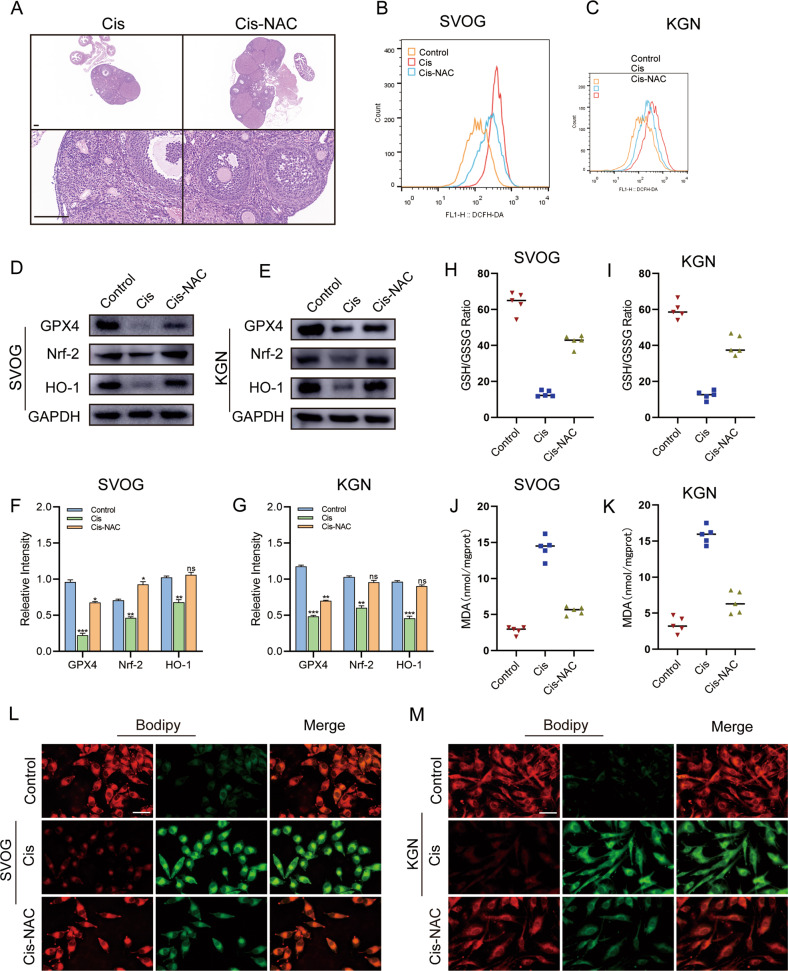
NAC inhibited ferroptosis by enhancing antioxidant capacity. **A** Representative images of H&E staining of ovarian micromorphology of mice after treatment with Cis or Cis + NAC. Scale bars = 200 μm. *n* = 5 (independent experiments). **B**, **C** ROS assay to evaluate the intracellular ROS levels of SVOG and KGN cells after Cis (25 μg/ml) or Cis +NAC (5 mM) treatments for 24 h. **D**, **E** The protein levels of GPX4, Nrf-2, and HO-1 were examined using Western blot analysis in SVOG (**D**) and KGN (**E**) cells treated with Cis (25 μg/ml) or Cis +NAC (5 mM) for 24 h. **F**, **G** Quantitative analysis of the targeted protein expressions. *n* = 3 (independent experiments). **H**, **I** The ratio of Intracellular GSH/GSSG was assayed after Cis (25 μg/ml) or Cis +NAC (5 mM) treatment for 24 h in SVOG (**H**) and KGN (**I**) cells. *n* = 5 (independent experiments). **J**, **K** Intracellular MDA were assayed after Cis (25 μg/ml) or Cis +NAC (5 mM) treatment for 24 h in SVOG (**J**) and KGN (**K**) cells. *n* = 5 (independent experiments). **L**, **M** Representative images of BODIPY staining indicated lipid ROS level after Cis (25 μg/ml) or Cis + NAC (5 mM) treatments (red for reduction, green for oxidization) in SVOG (**L**) and KGN (**M**) cells. Scale bar = 10 μm. *n* = 3 (independent experiments). *p < 0.05, **p < 0.01, ***p < 0.001. NS: not significant.

As a sulfhydryl donor, NAC can scavenge oxygen free radicals and play a direct role in antioxidants [20]. Thus, NAC was given orally to mice to investigate the role of NAC in Cis-induced ovarian damage after Cis treatment. As shown in Fig. 7A and S3, oral administration of NAC significantly improved ovarian volume and the number of antral follicles in Cis-treated mice. Subsequently, NAC treatment remarkably inhibited cellular ROS production in Cis-treated cells and considerably upregulated the cellular expression of GPX4, Nrf2, and HO-1 (Fig. 7B–G). Moreover, as a precursor of GSH, NAC is also the prerequisite for synthesizing intracellular GSH, which plays a major anti-oxidative role in cells (Fig. 7H, I). As expected, NAC treatment significantly upregulated the GSH/GSSG ratio in the Cis-treated ovarian GCs and downregulated MDA levels and protected the Cis-treated cells against excessive lipid ROS accumulation (Fig. 7J–M). Consequently, the abovementioned results suggested that NAC could improve Cis-induced ovarian damage by enhancing antioxidant capacity and inhibiting ferroptosis.

## Discussion

Accompanied by the diversification of therapeutic approaches, it is common for cancer survivors to own ever-increasing life spans, raising concerns about their quality of life after cancer treatment. An update on female breast cancer statistics in the United States released by the American Cancer Society states that between 1989 and 2020, breast cancer mortality dropped by 43% [21]. Simultaneously, the five-year relative survival rate respectively increased from 75% and 50% (diagnosed in the mid-1970s) to 90% and 65% (diagnosed from 2011 through 2017) for breast and colorectal cancer patients, which reflects both the advances in surgical techniques and accurate chemoradiotherapy [22]. Generally, chemotherapeutic drugs were applied to damage the viability of cells with high-proliferative capacity, which is a typical characteristic of cancer cells, but regrettably, they also induce damage to normal tissues and cause cardiotoxicity, gastrointestinal reaction, nephrotoxicity, peripheral nerve toxicity, and ovarian toxicity [23–27]. Of these, ovarian toxicity, the main side effect of curative chemotherapy, is notable and prevalent in female cancer patients. These long-term chemotherapy-induced ovarian damage mainly include but are not limited to decreased ovarian reserve, infertility, and ovarian atrophy. Therefore, exploring the underlying mechanism of chemotherapeutic drugs-induced ovarian damage will pave the way to develop fertility-protective adjuvants for female patients during conventional cancer treatment.

Depending on a series of experiments in vitro and in vivo, our present study confirmed the side effects of conventional chemotherapeutic drugs on ovarian morphology and function; further emphatically determined the ferroptosis involved in Cis-induced ovarian dysfunction. Not surprisingly, CTX, Tax, Dox, and Cis treatment significantly decreased the ovarian volume of mice and the number of primordial and antral follicles in the ovary; after that, various functional experiments were performed to verify their toxicity in ovarian fibrosis, apoptosis, receptivity, and function. Subsequently, Tax, Dox, and Cis treatment can induce the apoptosis of ovarian GCs, which is likely to result from excessive ROS production-induced oxidative damage and impaired anti-oxidative capacity. Thirdly, further experiments demonstrated that Cis treatment could induce mitochondrial dysfunction through overproducing superoxide in GCs and trigger lipid peroxidation leading to ferroptosis, first reported in chemotherapeutic drugs-induced ovarian damage. In addition, NAC treatment could alleviate the Cis-induced toxicity in GCs by downregulating cellular ROS levels and enhancing the anti-oxidative capacity (promoting the expression of GPX4, Nrf2, and HO-1).

Ovary, the key endocrine organ of the female reproductive system, is responsible for the birth of new life and secrete various hormones, such as anti-Müllerian hormone (AMH), estrogen, progesterone, and testosterone, exhibiting vital roles in maintaining the endocrinological homeostasis [28]. The ovary is also the main target attacked by chemotherapeutic drugs due to the periodic follicular development and highly-proliferative ovarian cells, especially in reproductive age [29]. Moreover, this ovarian damage causes fertility problems and increases the risk of cardiovascular disease and osteoporosis [30, 31]. As estimated, the patients with breast cancer who received 4 or more cycles of chemotherapy exhibited decreased serum estrogen levels and increased serum FSH levels reflecting the disordered ovarian function compared to that in the general population with correspondent age.

Apart from the disordered hormone secretion, several preclinical studies have confirmed the ovarian damage caused by chemotherapy. Dox can not only cause massive double-strand-DNA-breaks in a dose-dependent manner in primordial follicles, oocytes, and granulosa cells but also negatively affect ovarian vasculature and stromal tissue [32–34]. Simultaneously, CTX can promote the proliferation of the dormant primordial follicle population, leading to ovarian reserve depletion [2]. Another report also showed that Cis-treated ovaries manifested oocyte-specific damage [35]. Following these published studies, our results confirmed that the number of ovarian follicles at all stages in chemotherapeutic drugs-treated mice was significantly reduced, and no mature follicles were observed. Simultaneously, the subsequent examination further demonstrated that chemotherapeutic drugs in this study significantly aggravated ovarian fibrosis, promoted ovarian cell apoptosis, and decreased ovarian receptivity, leading to ovarian damage (morphological abnormality and dysfunction).

Consequently, understanding the mechanisms underlying the adverse effects of chemotherapy on ovarian function will pave the way to explore the potential fertility-preserving adjuvants that could improve the life quality of cancer patients. Over the past decades, several excellent reviews have summarized that chemotherapeutic drugs can directly toxify ovarian cells via impairing DNA replication and indirectly induce ovarian toxicity mediated by an acute vascular insult [17, 36, 37]. Notably, oxidative stress induced by chemotherapy is likely to negatively affect mitochondrial function and interfere with the dynamic balance of pro- and anti-apoptotic molecules in ovarian cells, leading to damage to different target cells, but the exact mechanism through which this occurs is still uncertain.

As a key participant in oxidative stress, ROS is involved in the cellular information transfer via a variety of signaling pathways, but excessive ROS production will damage cellular proteins, lipids, and DNA, resulting in fatal injury and even apoptosis in cells through both the extrinsic and intrinsic pathways [38, 39]. Simultaneously, ROS affects the activity of the mitochondrial respiratory chain complex, thus affecting its oxidative phosphorylation, reducing the synthesis of cellular ATP, and further promoting ROS production after sequential reactions [40]. Once the excessive ROS cannot be eliminated in time, it will likely result in a vicious cycle that continuously damages mitochondrial viability until irreversible apoptosis occurs [39]. Previous studies reported that the primary target of cisplatin-induced oxidative stress is the mitochondrion, resulting in loss of the sulfhydryl group on mitochondrial proteins, impairment of calcium uptake, and downregulation of mitochondrial membrane potential [7, 41, 42]. Similarly, Dox treatment has also been demonstrated to generate adducts by reacting with mitochondrial DNA that disturbs normal mitochondrial activity [43]. Another study indicated that complex IV was the primary site of Tax-induced mitochondrial ROS formation [44]. Our results confirmed that using Tax or Cis significantly induced excessive ROS production and damaged the mitochondrial membrane potential in ovarian GCs, leading to the impairment and even collapse of cellular antioxidant capacity reflected by the downregulation of cellular Nrf2 and HO-1 expression, as well as anti-apoptotic indicator Bcl-2.

RNA-seq was performed in the ovarian tissues between the mice received with and without Cis treatment to explore further the underlying outcome resulting from chemotherapy-induced disordered redox homeostasis. Although Cis is generally considered to have moderate ovarian toxicity, it exhibited the highest in our study, which is likely to correlate with its dosage. It is well-known that Cis can not only inhibit cell proliferation through DNA damage, particularly to mitotically-active cells, resulting in the activation of apoptotic cascades through the p53 signaling pathways[45], but also induce the reduction of glycolysis, dysfunction of the mitochondria, and excessive production of ROS [46–48]. Interestingly, RNA-Seq results first suggested that Cis-induced ovarian damage might result from ferroptosis, which closely correlates with excessive ROS production, although several studies have confirmed that ferroptosis was involved in chemotherapy-induced cancer cell death [49].

Ferroptosis refers to an iron-dependent mode of cell death caused by overloaded lipid peroxidation on cellular membranes and the mass accumulation of cellular ROS, which is morphologically and mechanistically distinct from typical apoptosis [50]. Cis could quickly bind to thiol-containing antioxidant molecules like glutathione and is transformed into a highly reactive state; when glutathione levels fall, intracellular oxidative stress rises, resulting in lipid peroxidation [51, 52]. Further, as a byproduct of lipid peroxidation, MDA levels were also reported to be significantly elevated by Cis [53]. Meanwhile, as an important cofactor for numerous metabolic enzymes, iron stimulates oxygen-and redox-based metabolism and the production of cellular ROS by acting as a catalyst for the Fenton reaction, which produces excessive lipid peroxidation [54, 55].

**Fig. 8:**
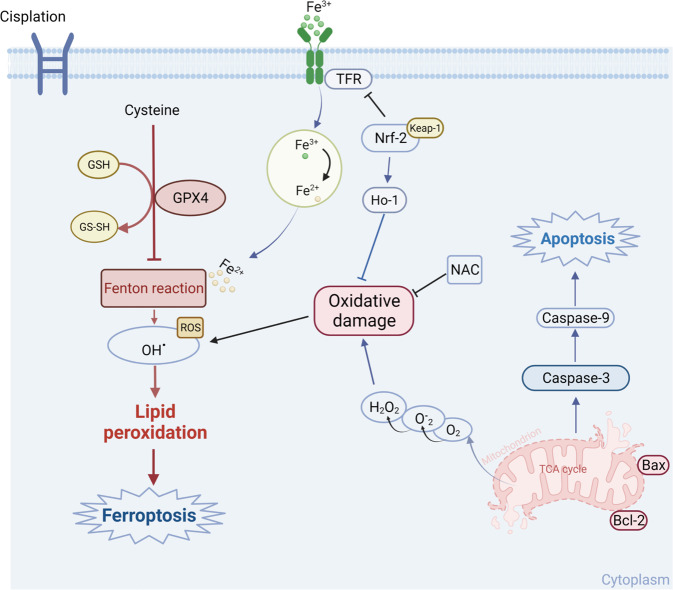
Potential mechanism by which Cis exacerbates ovarian GCs death. Exposed to Cis leads to ovarian GCs ferroptosis and apoptosis. Cis induces mitochondrial dysfunction and downregulates the expression of GPX4 in ovarian GCs, leading to excessive ROS production and upregulation of apoptosis-related factors. The excessive intracellular ROS combines with Fe^2+^ to initiate the Fenton reaction, thereby inducing cell ferroptosis. NAC rescues Cis-induced cell death by reducing excessive ROS levels and enhancing antioxidant capacity.

Therefore, based on our abovementioned results in vitro and in vivo, we reasonably hypothesized that Cis-induced disordered oxidative stress (excessive ROS production) could activate the ferroptosis-associated signaling pathway in ovarian cells, leading to irreversible ovarian damage (Fig. 8). Ovarian GCs were selected for the subsequent examinations to test the above hypothesis. As expected, our results completely fulfilled the characteristics of ferroptosis in Cis-treated GCs. Both the significant downregulation of GSH, GPX4, and upregulation of MDA, TFR, and lipid peroxidation were observed. Simultaneously, lipid peroxidation is primarily generated during multiple steps of cellular metabolism, in which mitochondrion plays a central role. Therefore, ferroptosis is strongly linked with a dramatic change in mitochondrial morphology, mainly including fragmentation and cristae disappearance [56]. Our study also demonstrated that in Cis-treated GCs, Cis first causes mitochondrial membrane potential hyperpolarization and generates excessive mitochondrial ROS. Then, with the time extension of Cis treatment in GCs, mitochondrial membrane potential depolarization, mitochondrial swelling, rupture, and crista disappearance sequentially occurred, resulting in cellular ferroptosis.

Similarly, previous studies have demonstrated that Cis increases renal iron levels while promoting iron-catalyzed oxidative damage and renal ferroptosis [57]. Although chemotherapeutic drugs can also induce ferroptosis in cancer cells, the sensitivity to chemotherapeutic drugs-induced ferroptosis between cancer cells and normal cells is inconsistent [58]. Cancer cells exhibit a high tolerance and resistance to chemotherapeutic drugs-induced ferroptosis, which should be enhanced in antitumor therapies [59]. Instead, normal cells exhibit high sensitivity and fragility to chemotherapeutic drugs-induced ferroptosis, which should be ameliorated if chemotherapy is inevitable [60].

Overall, the final aim of illustrating the specific mechanism under chemotherapy-induced ovarian damage is to identify fertility-protective adjuvants that can be targeted to block the negative response to chemotherapeutic drugs, thus preserving as many ovarian functions as possible during cancer treatment. Unfortunately, as the main candidate for preserving ovarian function during chemotherapy, gonadotropin-releasing hormone agonist has been extensively verified in most randomized clinical trials, but with little to no data showing positive effects in women with hematological malignancies [61–63]. Based on the published results and our findings, developing the protectants from the perspective of chemotherapy-induced oxidative stress resulting from the imbalance between free radicals production and elimination is a good alternative. Promisingly, several antioxidants, including melatonin, resveratrol, and rutin, have exhibited the ability to maintain low ROS levels in vitro and in vivo and further contributed to improving ovarian reserve by preventing oxidative stress [64–66]. Based on the above-published reports, our study also confirmed that NAC, a conventional antioxidant ameliorated the cisplatin-induced ovarian damage in vitro and in vivo, which was likely to be contributed by reducing ROS levels and enhancing antioxidant capacity in ovarian cells and then inhibiting cellular ferroptosis.

In summary, the indispensability of chemotherapy in cancer treatment caused the inevitability of chemotherapy-induced ovarian damage in female cancer patients, and further exploring the underlying mechanism contributes to identifying appropriate fertility-protective adjuvants. Our study confirmed the chemotherapy-induced chaotic hormonal state and ovarian damage in the preclinical and clinical examination and indicated that chemotherapeutic drugs initiated ferroptosis in ovarian cells through excessive ROS-induced lipid peroxidation and mitochondrial dysfunction, leading to ovarian cell death. Consequently, developing fertility protectants from the chemotherapy-induced oxidative stress and ferroptosis perspective will ameliorate ovarian damage and further improve the life quality of cancer patients.

## Supplementary information

Chemotherapeutic agents induced ovarian GCs injury.

Chemotherapeutic agents induced ovarian GCs dysfunction.

NAC rescued follicle loss caused by Cis.

Abnormal gonadal hormone levels in patients who received chemotherapy.

Details of antibodies used.

Supplementary legends

Original Data

A reproducibility checklist that details key elements of the experimental and analytical design of the submission.

## Data Availability

The raw data of high-throughput RNA-sequencing analysis have been deposited to the Sequence Read Archive (SRA) database, produced by the National Center for Biotechnology Information (NCBI) at the National Institutes of Health (NIH), with the BioProject accession number PRJNA940205.
