# Supplementary figures and images for: Chemotherapy impairs ovarian function through excessive ROS-induced ferroptosis
Source: Cell Death Dis. 2023 May 24;14(5):340. doi: 10.1038/s41419-023-05859-0 (PMC10209065; doi:10.1038/s41419-023-05859-0)

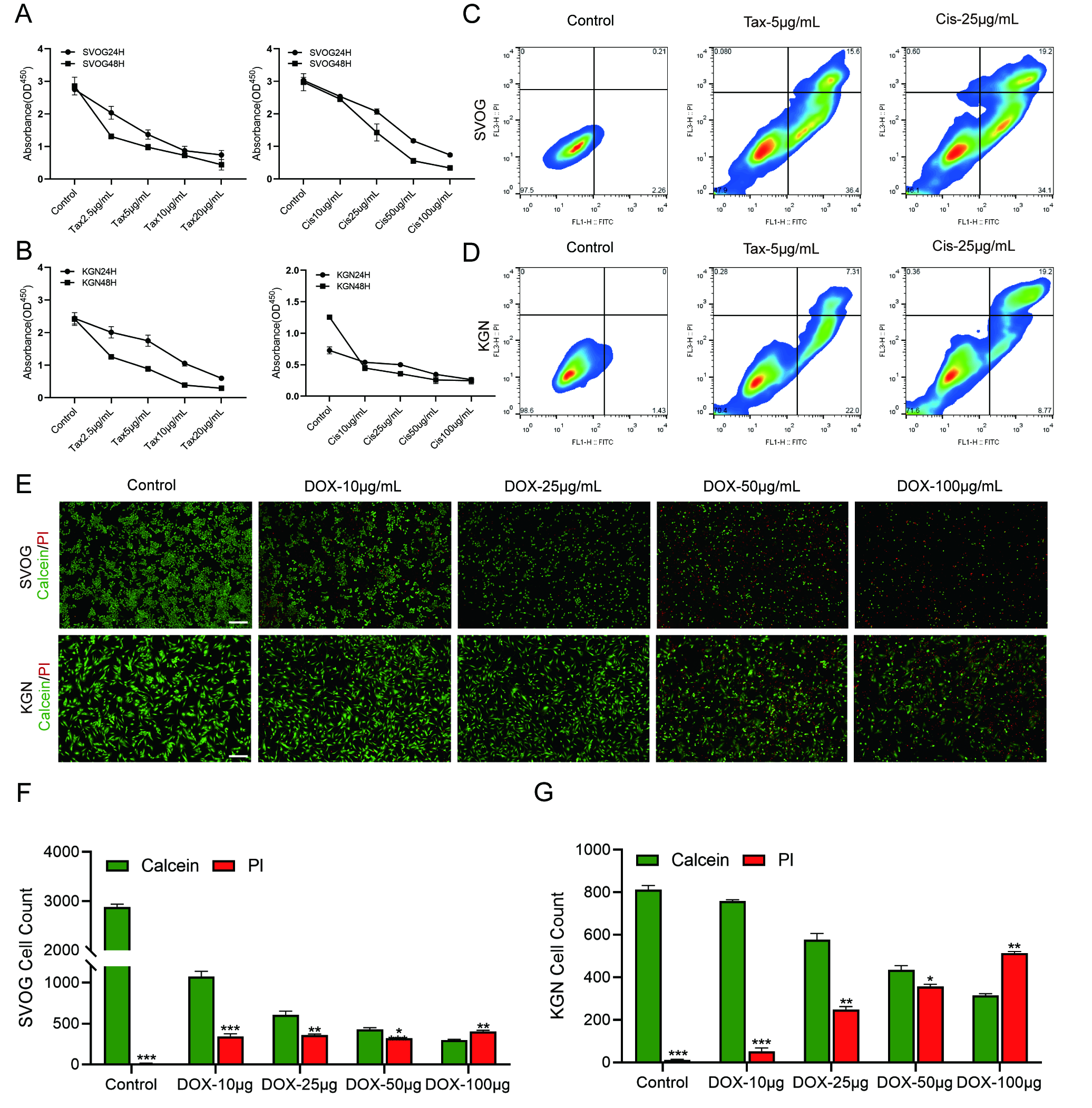

Supplement: Supplementary file 1 — Chemotherapeutic agents induced ovarian GCs injury. [file 41419_2023_5859_MOESM1_ESM.tif]

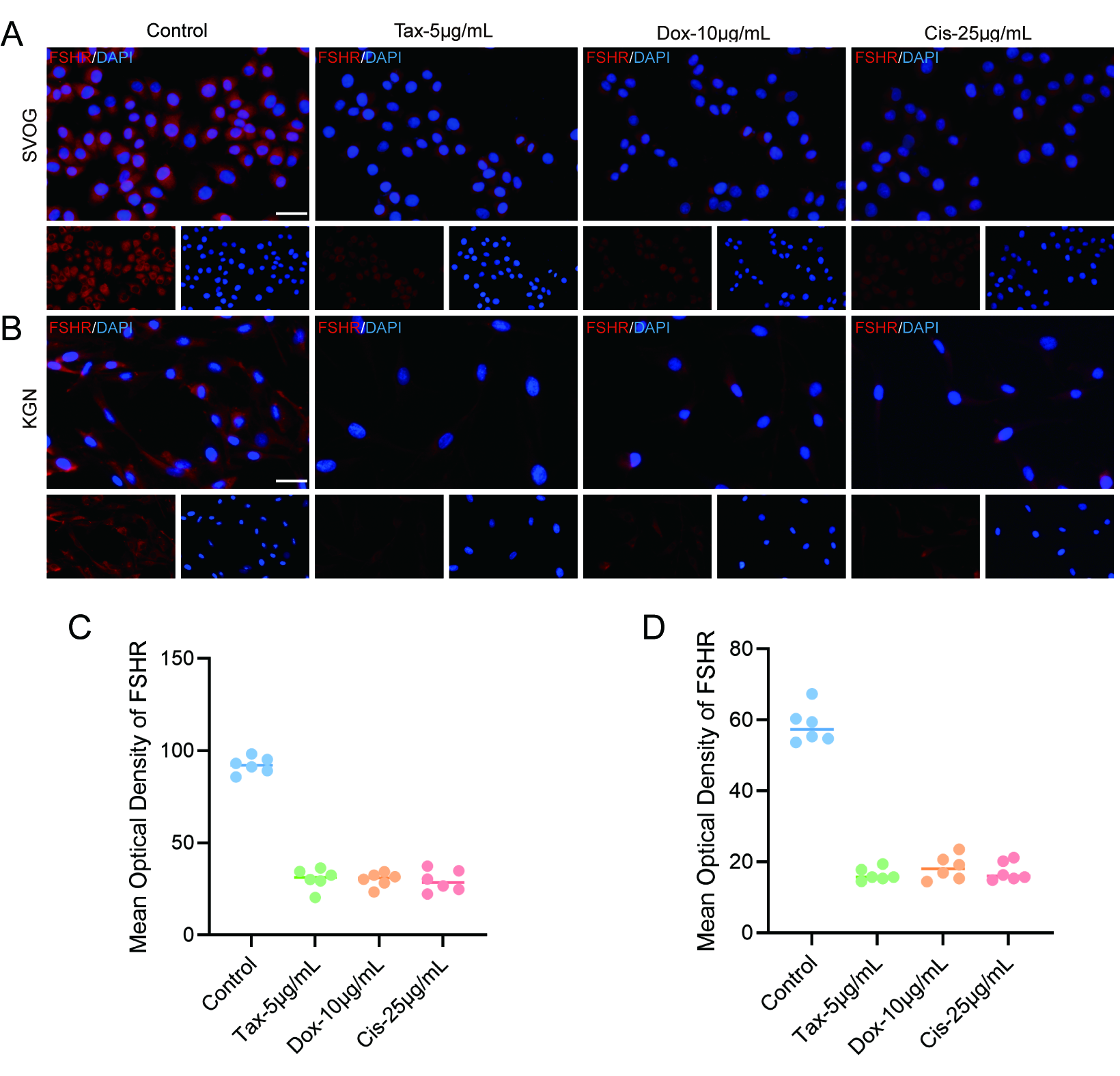

Supplement: Supplementary file 2 — Chemotherapeutic agents induced ovarian GCs dysfunction. [file 41419_2023_5859_MOESM2_ESM.tif]

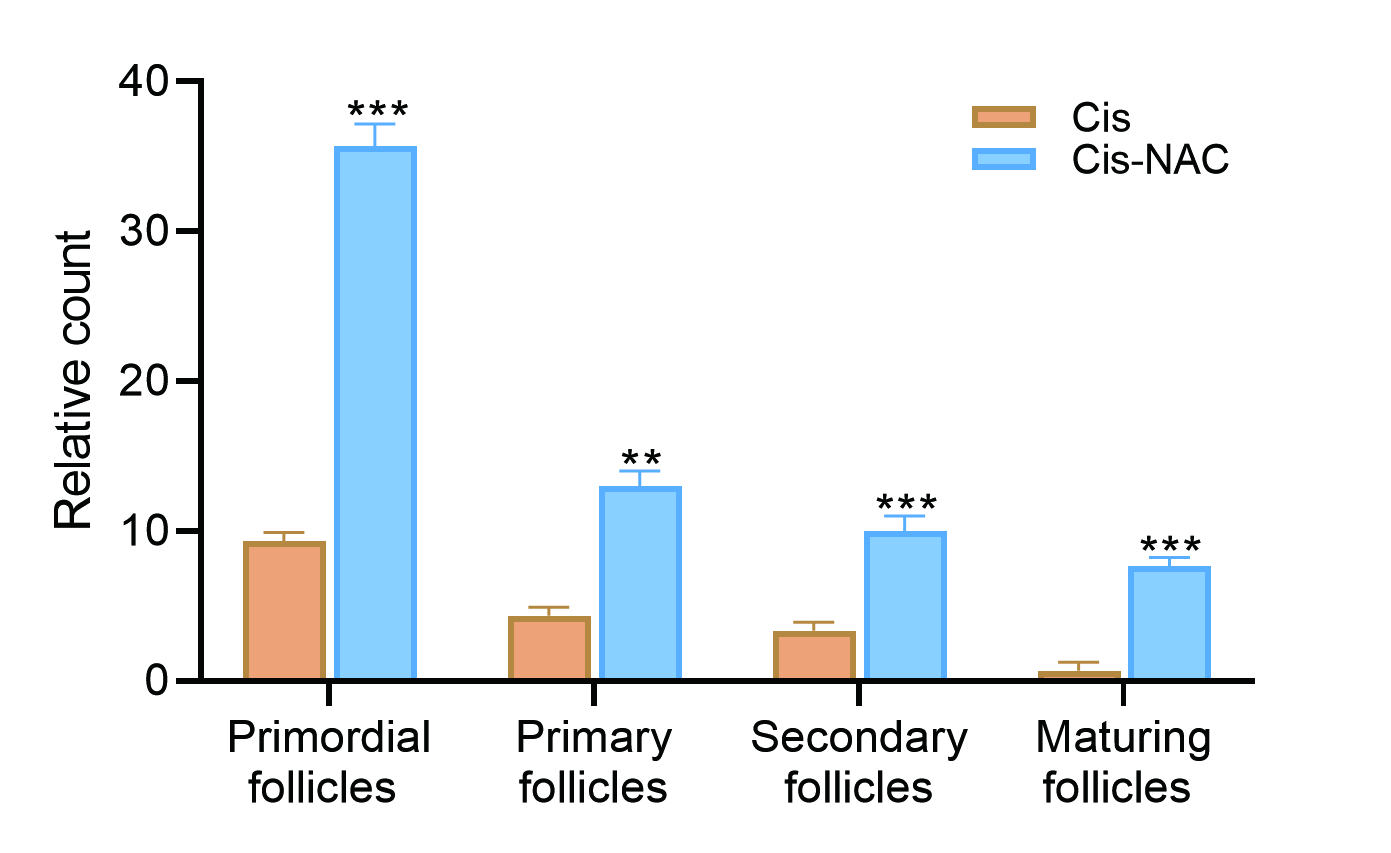

Supplement: Supplementary file 3 — NAC rescued follicle loss caused by Cis. [file 41419_2023_5859_MOESM3_ESM.tif]
