# Supplementary material for: Chemotherapy impairs ovarian function through excessive ROS-induced ferroptosis
Source: Cell Death Dis. 2023 May 24;14(5):340. doi: 10.1038/s41419-023-05859-0 (PMC10209065; doi:10.1038/s41419-023-05859-0)
Supplement: Supplementary file 5 — Details of antibodies used. [file 41419_2023_5859_MOESM5_ESM.docx]

**Table S2 Details of antibodies used**

| **Antibodies** | **Manufacturer** | **Catalog No** | **Test** | **Concentration used** |
| --- | --- | --- | --- | --- |
| AMH Polyclonal antibody | Proteintech | 14461-1-AP | IHC/WB | 1:100/1:1000 |
| TGF-β Antibody | CST | 3711 | WB | 1:1000 |
| FSHR Antibody | Affinity | DF4927 | WB | 1:1000 |
| KEAP1 (D6B12) Rabbit mAb | CST | 8047 | WB | 1:1000 |
| NRF2 (D1Z9C) XP®Rabbit mAb | CST | 12721 | WB | 1:1000 |
| HO-1 (D60G11) Rabbit mAb | CST | 5853 | WB | 1:1000 |
| Cleaved Caspase-3 (Asp175) Antibody | CST | 9661 | WB | 1:1000 |
| Bcl-2 Antibody | Affinity | AF6139 | WB | 1:1000 |
| GPX4 Antibody | Affinity | DF6701 | IF/WB | 1:50/1:1000 |
| Transferrin Receptor Antibody | Affinity | AF5343 | WB | 1:1000 |
| GAPDH Antibody | Abways | AB0037 | WB | 1:1000 |
| Goat anti-rabbit IgG-HRP | absin | abs20040 | WB | 1:5000 |
| Goat anti-Mouse IgG-AF594 | absin | abs20146 | WB | 1:5000 |
| ER-α Antibody | Servicebio | GB13205 | IF | 1:100 |
| Anti -FSH-R Rabbit pAb | Servicebio | GB11275-1 | IF | 1:200 |
| Goat Anti-Rabbit IgG H&L (HRP) | Servicebio | GB23303 | IF | 1:500 |
| Alexa Fluor® 488-conjugated Goat Anti-Rabbit IgG (H+L) | Servicebio | GB25303 | IF | 1:200 |
| CY3-Tyramide | Servicebio | G1223 | IF | 1:500 |
