# Supplementary material for: Chemotherapy impairs ovarian function through excessive ROS-induced ferroptosis
Source: Cell Death Dis. 2023 May 24;14(5):340. doi: 10.1038/s41419-023-05859-0 (PMC10209065; doi:10.1038/s41419-023-05859-0)
Supplement: Supplementary file 6 — Supplementary legends [file 41419_2023_5859_MOESM6_ESM.docx]

**Fig. S1. Chemotherapeutic agents induced ovarian GCs injury. A, B.** CCK-8 assay to measure SVOG and KGN cell viability after (0, 2.5, 5, 10, 20 μg/ml) or Cis (0, 10, 25, 50, 100 μg/ml) treatments for 24 or 48 hours. n=3 (independent experiments). **C, D.** SVOG, and KGN cells treated with Tax (5 μg/ml) or Cis (25 μg/ml) for 24 h and then stained with annexin V-FITC/PI were determined using flow cytometry. **E-G.** Live/Dead assay staining for SVOG and KGN cells with DOX (0, 10, 25, 50, 100μg/ml) treatment (green for live cells, red for dead cells). Scale bar = 100 μm. The Live/Dead number of cells was quantified for SVOG (**F**) and KGN (**G**) cells. n=3 (independent experiments).

**Fig. S2. Chemotherapeutic agents induced ovarian GCs dysfunction. A-D.** Representative immunofluorescence images of FSHR in the SVOG **(A)** and KGN **(B)** cells after Tax (5 μg/ml), Dox (10 μg/ml), and Cis (25 μg/ml) treatment were shown. Scale bar =10 μm. The quantified fluorescence intensity of FSHR in SVOG **(C)** and KGN **(D)** cells were shown. n=6 (independent experiments).

**Fig. S3. NAC rescued follicle loss caused by Cis.** The quantities of primordial, primary, secondary, and maturing follicles per slide in each group were analyzed. n=5 (independent experiments). **p* < 0.05, ***p* < 0.01, ****p* < 0.001.

**Table. S1. Abnormal gonadal hormone levels in patients who received chemotherapy.**

**Table. S2. Details of antibodies used.**
