# Supplementary material for: Chemotherapy impairs ovarian function through excessive ROS-induced ferroptosis
Source: Cell Death Dis. 2023 May 24;14(5):340. doi: 10.1038/s41419-023-05859-0 (PMC10209065; doi:10.1038/s41419-023-05859-0)

Fig. 3

AMH

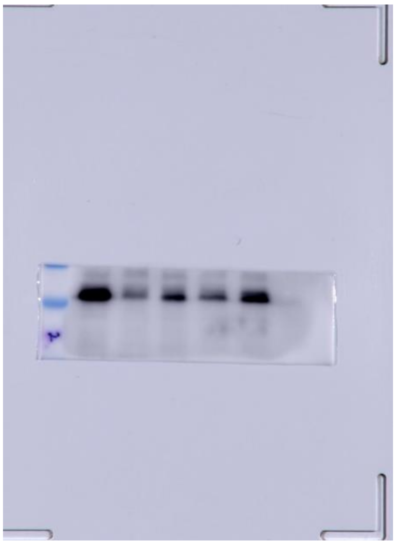

FSHR

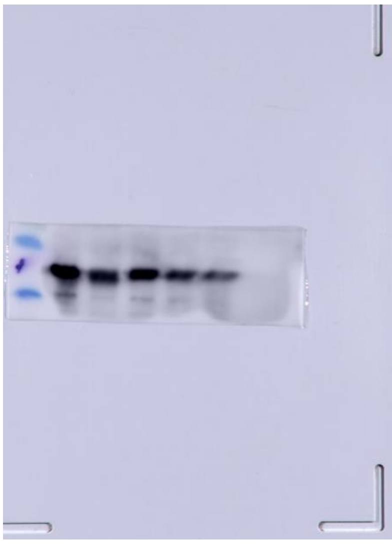

GAPDH

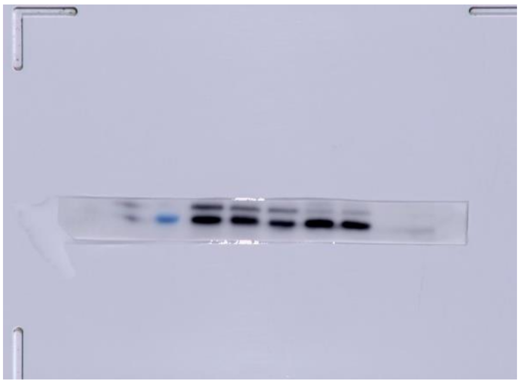

TGF-β

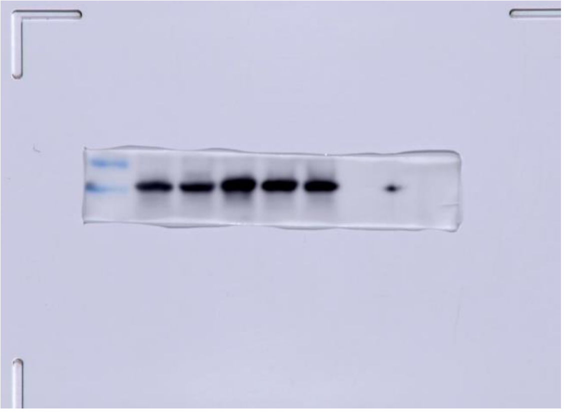

Fig. 4
KGN
Bcl-2

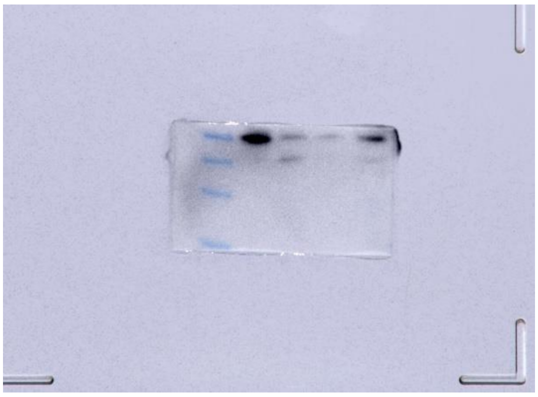

Cleaved caspase-3

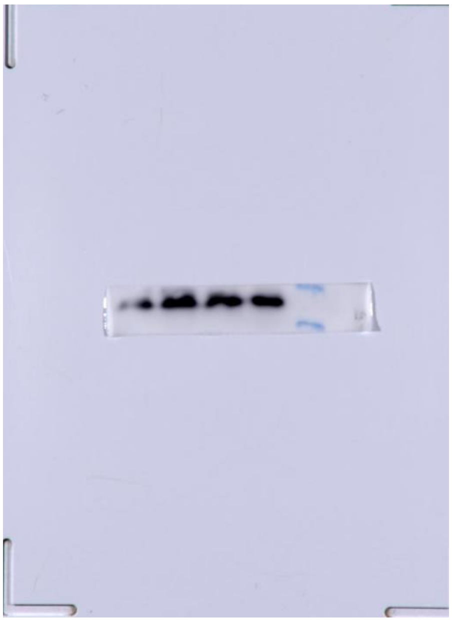

HO-1

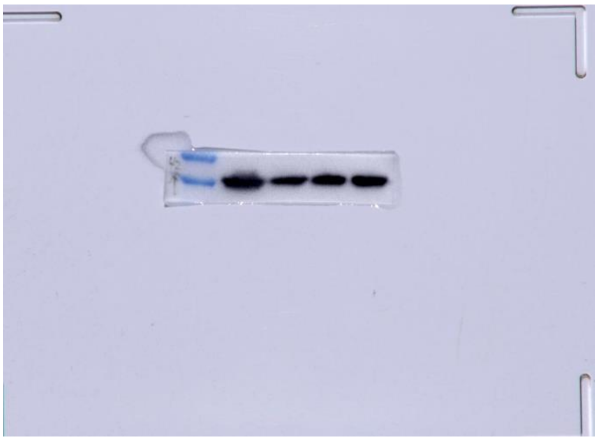

Keap-1

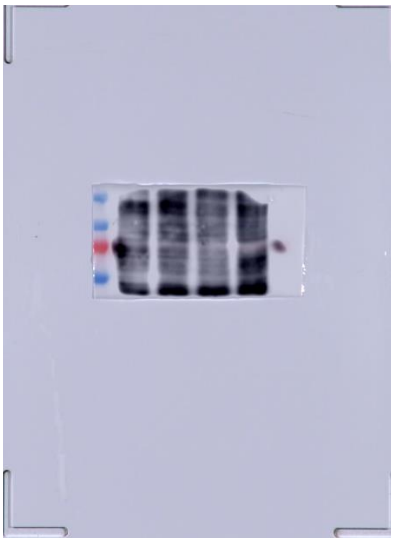

Nrf-2

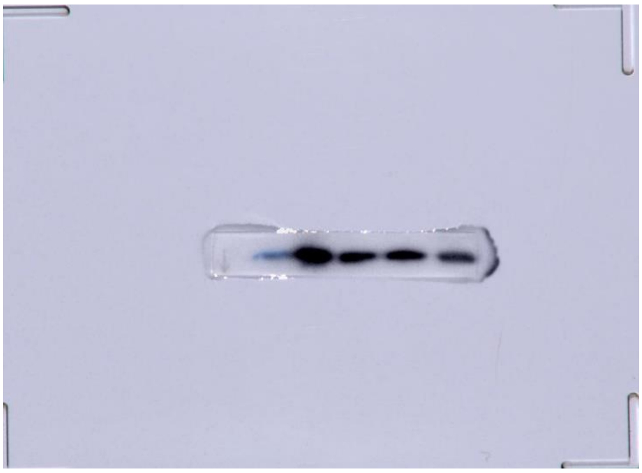

GAPDH

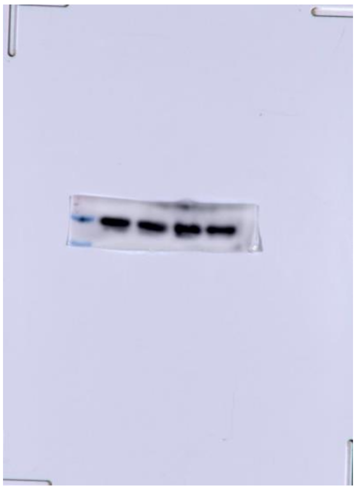

SVOG
Bcl-2

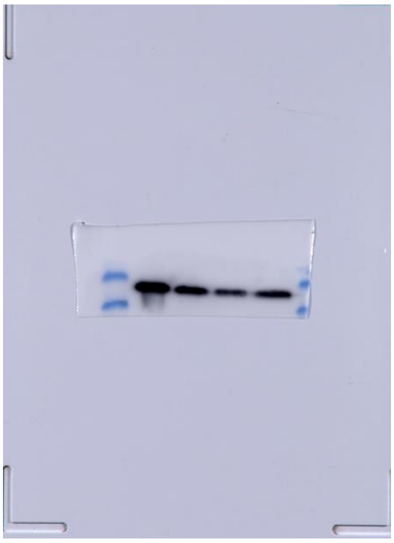

CLeaved-Caspase-3

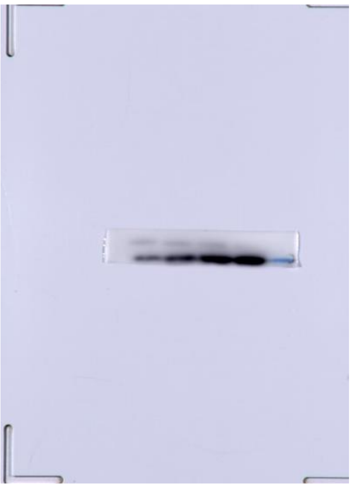

HO-1

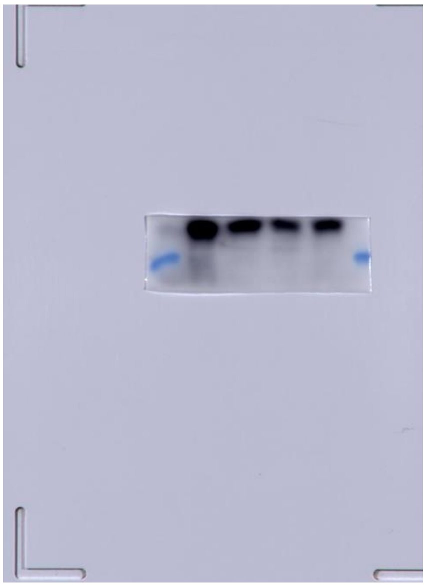

Keap-1

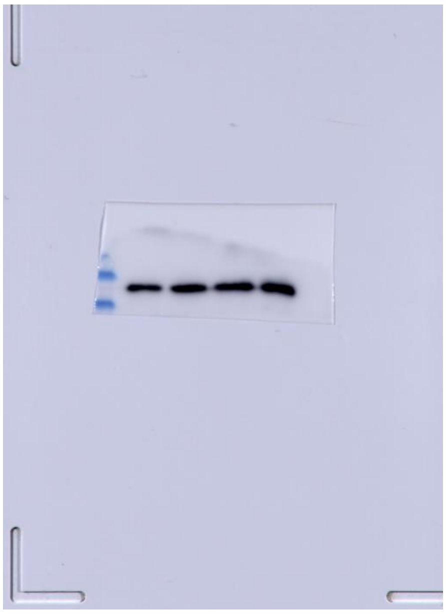

Nrf-2

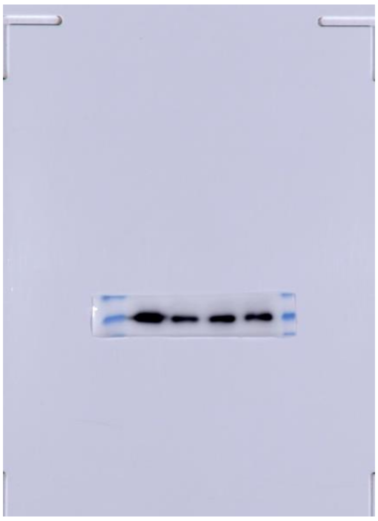

GAPDH

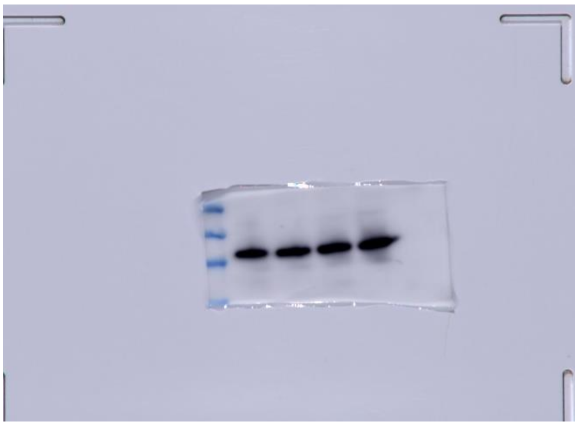

KGN

GPX4

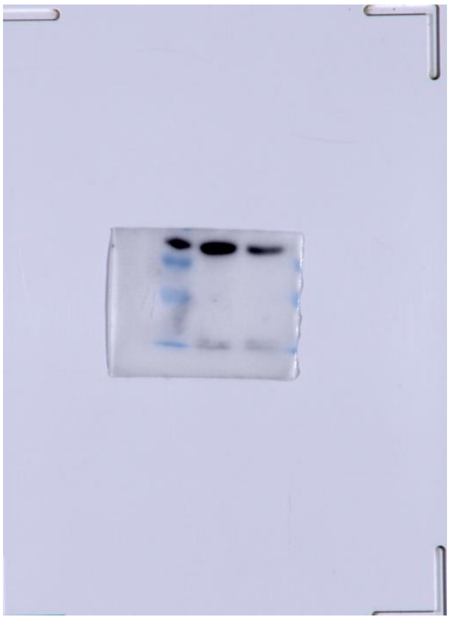

Nrf-2

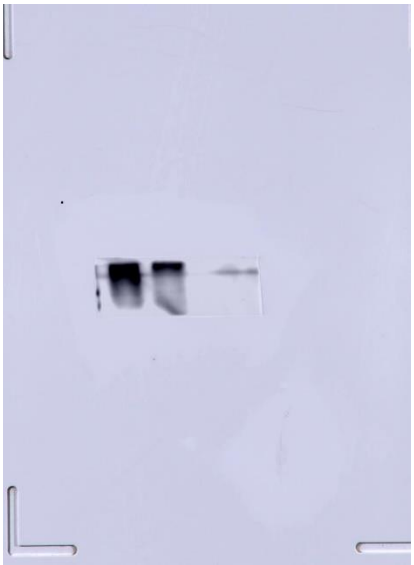

TFR

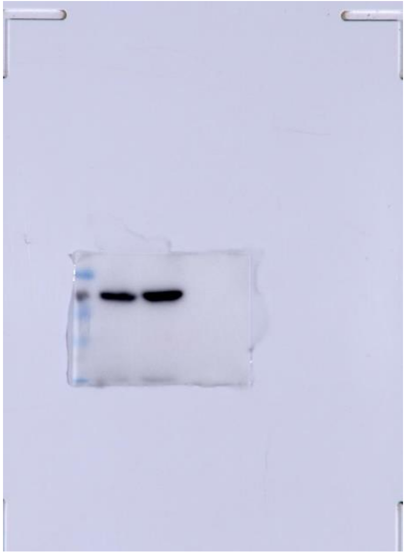

GAPDH

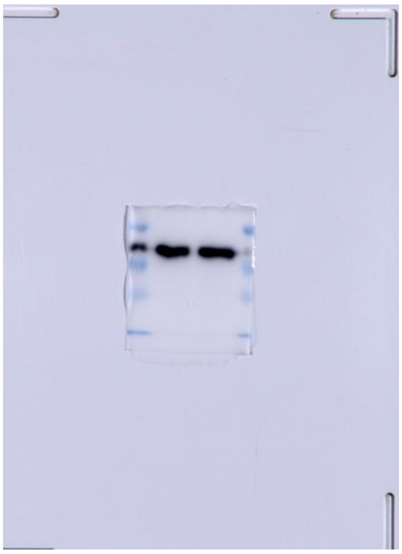

SVOG

GPX4

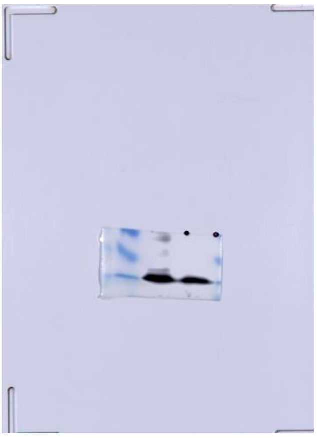

Nrf2

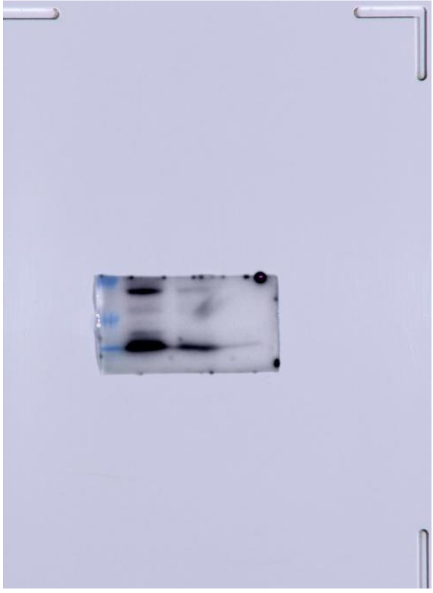

TFR

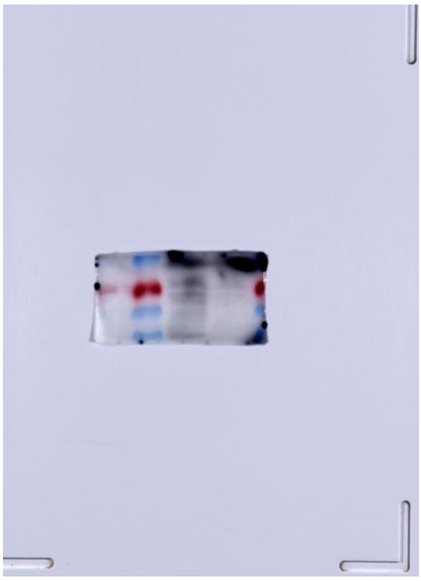

KGN

GPX4

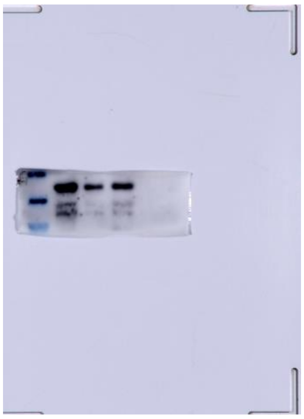

HO-1

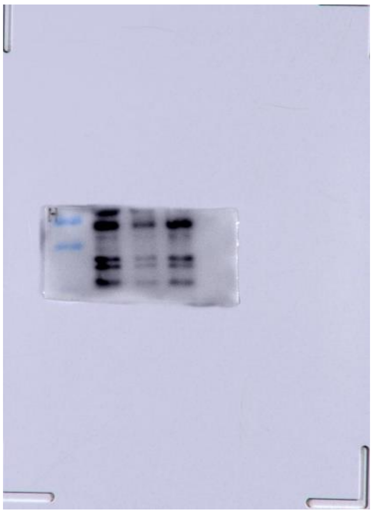

Nrf-2

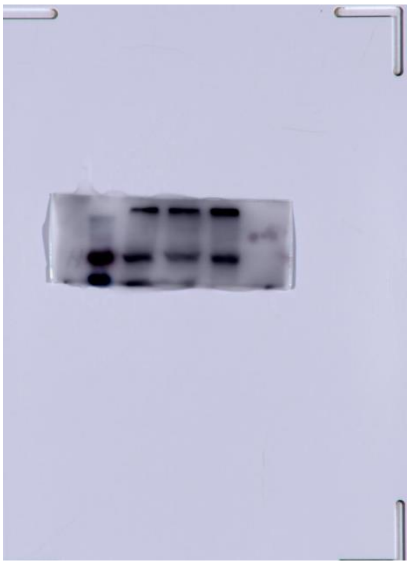

GAPDH

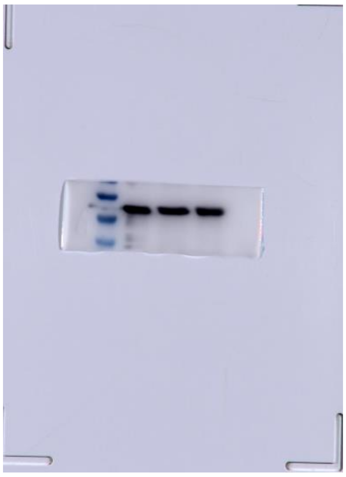

SVOG

GPX4

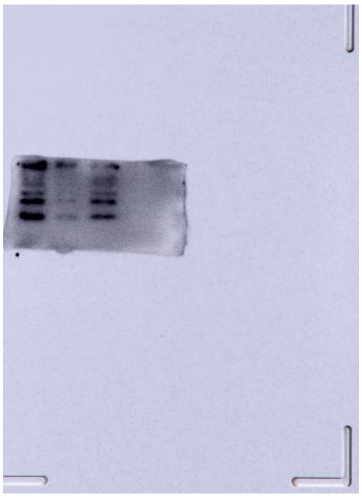

HO-1

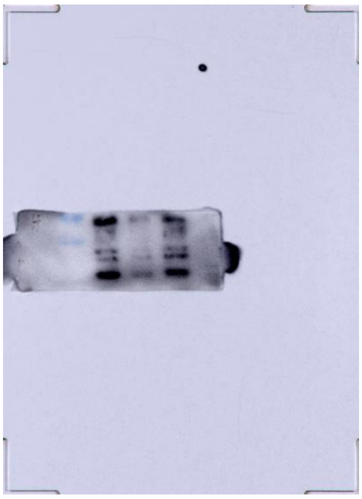

Nrf-2

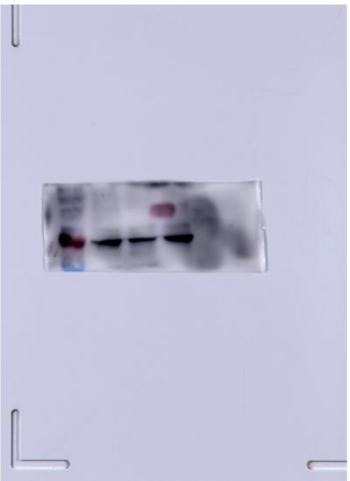

GAPDH

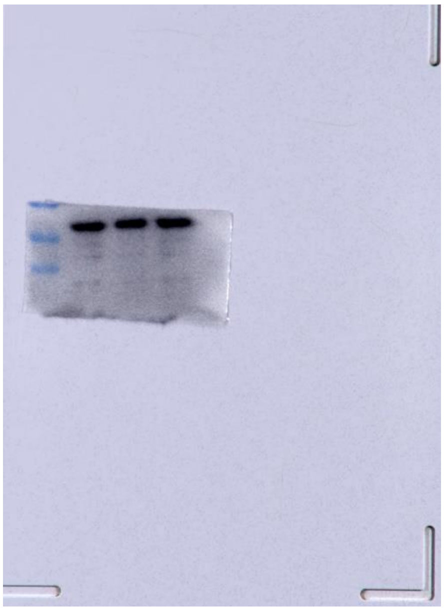

Supplement: Supplementary file 7 — Original Data [file 41419_2023_5859_MOESM7_ESM.pdf]
